# Spectral Asymmetry and Higuchi's Fractal Dimension Measures of Depression Electroencephalogram

**DOI:** 10.1155/2013/251638

**Published:** 2013-10-22

**Authors:** Maie Bachmann, Jaanus Lass, Anna Suhhova, Hiie Hinrikus

**Affiliations:** Department of Biomedical Engineering, Technomedicum of the Tallinn University of Technology, 5 Ehitajate Road, 19086 Tallinn, Estonia

## Abstract

This study was aimed to compare two electroencephalogram (EEG) analysis methods, spectral asymmetry index (SASI) and Higuchi's fractal dimension (HFD), for detection of depression. Linear SASI method is based on evaluation of the balance of powers in two EEG frequency bands in one channel selected higher and lower than the alpha band spectrum maximum. Nonlinear HFD method calculates fractal dimension directly in the time domain. The resting EEG signals of 17 depressive patients and 17 control subjects were used as a database for calculations. SASI values were positive for depressive and negative for control group (*P* < 0.05). SASI provided the true detection rate of 88% in the depressive and 82% in the control group. The calculated HFD values detected a small (3%) increase with depression (*P* < 0.05). HFD provided the true detection rate of 94% in the depressive group and 76% in the control group. The rate of correct indication in the both groups was 85% using SASI or HFD. Statistically significant variations were not revealed between hemispheres (*P* > 0.05). The results indicated that the linear EEG analysis method SASI and the nonlinear HFD method both demonstrated a good sensitivity for detection of characteristic features of depression in a single-channel EEG.

## 1. Introduction

Mental disorders are widespread in the population. According to statistics by National Institute of Mental Health, approximately one quarter of adults are diagnosable for one or more disorders in the United States [1]. Major depressive disorder is one of the most common mental disorders: about 6.7% of population suffers from depression, and the rate is increasing [2].

Nowadays, the diagnosis of depression is based mainly on evaluation of the intensity of subjective symptoms using questionnaire and interview. On the other hand, any declinations in the brain functioning are expected to be reflected in the brain bioelectrical activity. Therefore, the electroencephalography (EEG) is a valuable method for getting objective information about the changes in brain physiology specific for depression. 

Brain behaves as a complex nonlinear system [3–5]. Therefore, nonlinear methods for analysis of EEG signal are expected to provide more information about properties of the brain compared to linear methods. Various linear and nonlinear methods have been used in EEG analysis, as higher order spectra, recurrence quantification analysis, entropy and complexity measures, cumulants, and so forth [5–8]. Nonlinear methods of EEG analysis as fractal dimension, correlation dimension, and detrended fluctuation analysis have been demonstrated to be effective in detecting special EEG features in brain related to epilepsy, schizophrenia and Alzheimer's disease by many authors [9–13]. 

In the analysis of depression EEG mostly linear methods have been used. Specific features of resting EEG in depression as changes in EEG bands powers and frontal interhemispheric asymmetry have been reported by several authors [14–16]. Discriminant analysis of quantitative EEG classified correctly 91.3% of the patients and controls [14]. However, the validity potential of frontal alpha asymmetry as a clinical measure for depression still remains unclear [17].

In our previous study, a measure for evaluation of depression based on resting EEG spectral frequency features was developed [18, 19]. The spectral asymmetry index (SASI) is based on evaluating balance of powers in two EEG frequency bands in one EEG channel selected higher and lower than the alpha band spectrum maximum [18, 19]. Compared to interhemispheric asymmetry and coherence, SASI provided better discrimination between depressive and healthy subjects [19].

Only few results have been reported on application of nonlinear methods for detecting specific properties of EEG related to depression [20, 21]. The study by Hosseinifard et al. shows that various methods of nonlinear EEG analysis, including detrended fluctuation analysis (DFA), Higuchi's fractal dimension (HFD), correlation dimension (CD), and Lyapunov exponent, each provide classification of depression. Application of combined nonlinear features provides better discrimination for depressed and normal subjects (90%) compared to EEG bands power (76.6%) [20]. Ahmadlou et al. concluded that Katz's fractal dimension revealed no meaningful difference in frontal EEG between depressive and control subjects, whereas Higuchi's fractal dimension was more informative [21]. The machine learning techniques classifiers with multichannel EEG data input were used for classification in these studies [20, 21].

On the other hand, in some recent clinical applications, correlation between EEG biomarkers and changes in depression mode throughout the treatment course was not revealed [22–24]. The reported analysis of EEG data from the study of transcranial magnetic stimulation (TMS) showed that EEG power in multiple bands measured at baseline and throughout the treatment course did not correlate with eventual response to TMS treatment [22]. During a neurofeedback session, it was tested if the balance between left and right frontal alpha activity could be changed; however, no changes in mood were observed [23]. Results of another study indicated a negative association between parietal-occipital alpha power in the eyes open resting state and depression severity estimated by the brain-derived neurotrophic factor as one of the important factors in the etiology of major depressive disorder [24]. The results reported in the cited above clinical studies confirm that the linear EEG analysis, based on the absolute level of EEG rhythms power, does not provide detecting alterations in depression severity throughout the treatment course [22–24]. Therefore, studies in selection of more sensitive dependable EEG analysis methods for detection of depression are required. 

Idea of this study was comparison of linear and nonlinear EEG analysis methods for discrimination of depression. The selection of methods for comparison was based firstly on the previous results achieved with the method and secondly on the simplicity of calculation algorithm. 

Our previous results demonstrated that linear SASI method provides higher sensitivity for detection of depression compared to other methods as interhemispheric asymmetry or coherence at the comparable complicacy of computing algorithms [19]. 

HFD has been shown to provide better discrimination (91.3%) compared to Katz's fractal dimension [21]. Comparison of CD, HFD and DFA for classification of depression indicated CD showing the highest sensitivity (classification 83.3%), while HFD and DFA sensitivity was somewhat lower (76.6%) [20]. However, HFD calculates fractal dimension of time series directly in the time domain, and the HFD algorithm is much more straightforward and faster compared to CD (and also DFA) calculation algorithms [25]. Therefore, SASI as a linear, and HFD as a nonlinear EEG analysis method were selected for comparison in the analysis of depression EEG in this study. 

## 2. Materials and Methods 

### 2.1. EEG Signals

The resting eyes closed EEG signals of 17 depressive patients and 17 control subjects were used as a database for calculations of spectral asymmetry index and Higuchi's fractal dimension. The group of depressive patients consisted of female subjects without antidepressant treatment, average age 39 years, with standard deviation 12 years. The control group included matched by age healthy female subjects. The EEG recordings were performed in electrically and acoustically shielded dim laboratory room to avoid external disturbances. The subjects under investigation were lying in relaxed position with closed eyes during the recordings.

**Figure 1:**
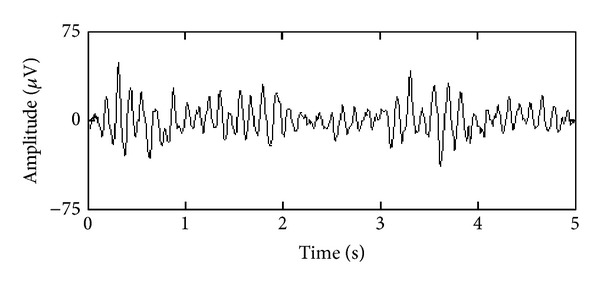
An example of the recorded depression EEG signal in channel P3.

The 5 min EEG signals were recorded from 18 channels according to the international 10-20-electrode position classification system. The Cadwell Easy II EEG measurement equipment was used for recording raw EEG signals with a frequency band of 0.3–70 Hz. The EEG signals were further filtered using Butterworth band-pass 0.5–48 Hz filter with an attenuation of 100 dB in the stopband and stored on a computer at the sampling frequency of 400 Hz. An example of the recorded EEG signal is presented in Figure 1. 

The study was conducted in accordance with the Declaration of Helsinki and approved by the Tallinn Medical Research Ethics Committee. 

EEG analysis was performed offline using Matlab software. SASI and HFD were calculated in frontal FP1 and FP2, temporal T3 and T4, parietal P3 and P4, and occipital O1 and O2 EEG channels using Cz as reference. 

### 2.2. Spectral Asymmetry Index Method—SASI

The principle of SASI is estimation of the spectral asymmetry of EEG spectrum regarding its maximum in alpha band [18]. For this purpose, the relative difference of the powers in the frequency bands selected higher and lower than alpha is calculated in one EEG channel. To achieve the balance of two powers in lower and higher EEG frequency bands, the width of the selected bands must compensate the difference in the EEG spectral density. The central (alpha) band frequencies are excluded from the analysis. The boundary frequencies selected for calculation frequency bands were adjusted taking into account the individual alpha for a subject. 

**Figure 2:**
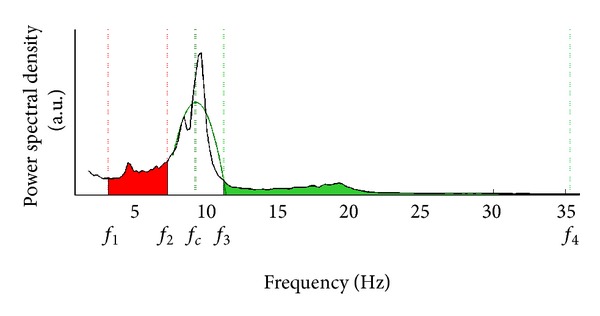
The scheme of SASI method: an EEG spectrum (black line) and a parabolic approximation (green line); *f*_*c*_—the maximum of parabolic approximation; *f*_1_ and *f*_2_—lower and higher boundary frequencies of the lower EEG frequency band; *f*_3_ and *f*_4_—lower and higher boundary frequencies of the higher EEG frequency band.

The principal scheme of the SASI method is presented in Figure 2. The boundary frequencies of the lower and higher frequency bands are related to the central frequency of the spectrum *f*_*c*_, located in alpha band. The lower frequency band was selected from *f*_1_ to *f*_2_, where

(1) $$\begin{array}{c} f_{1}=\left(f_{c}-6\right)\;\text{Hz},\;\;f_{2}=\left(f_{c}-2\right)\;\text{Hz}. \end{array}$$

The higher frequency band was selected from *f*_3_ to *f*_4_, where

(2) $$\begin{array}{c} f_{3}=\left(f_{c}+2\right)\;\text{Hz},\;\;f_{4}=\left(f_{c}+26\right)\;\text{Hz}. \end{array}$$

In this selection, the alpha-band width is presumed to be equal 4 Hz. The width of the lower frequency band is also selected as 4 Hz, close to traditional theta band. The higher frequency band is selected of 24 Hz covering EEG beta band frequencies. The selected bands are not necessarily matched to traditional EEG bands frequencies. The bandwidths were selected empirically during our previous studies [19]. 

The values of EEG powers in the frequency bands of interest, selected higher and lower of the spectrum maximum, were calculated for each EEG channel *m* and subject *n*. The power in the lower frequency band *P*_*Lmn*_ was calculated as

(3) $$\begin{array}{c} P_{Lmn}=\sum_{f=f_{1}}^{f_{2}}S{\left(f\right)}_{mn}, \end{array}$$

where frequencies *f*_1_ and *f*_2_ are determined by conditions (1) and *S*(*f*)_*mn*_ is power spectral density of the recorded EEG signal. The power in the higher frequency band *P*_*Hmn*_ was calculated as

(4) $$\begin{array}{c} P_{Hmn}=\sum_{f=f_{3}}^{f_{4}}S{\left(f\right)}_{mn}, \end{array}$$

where frequencies *f*_3_ and *f*_4_ are determined by conditions (2). The power spectral density of the signal *S*(*f*)_*mn*_ was calculated using Welch's averaged periodogram method. The signal was divided into series of overlapped segments (50% overlapping) with the length of 1024 samples. Every segment was multiplied by Hanning window function:

(5) $$\begin{array}{c} w\left(n\right)=0.5\left(1-\cos\frac{2\pi i}{N-1}\right), \end{array}$$

where *i* is a sample index and *N* is the number of samples in a segment. The periodogram was calculated by applying fast fourier transform to a windowed segment and time-averaging of the result. 

The general algorithm for calculation of SASI presents relative difference of EEG powers in the frequency bands selected higher and lower than alpha band frequencies:

(6) $$\begin{array}{c} {\text{SASI}}_{mn}=\frac{P_{Hmn}-P_{Lmn}}{P_{Hmn}+P_{Lmn}}, \end{array}$$

where *P*_*Hmn*_ is determined by (3) and *P*_*Lmn*_ by (4). 

The formula (6) provides differentiation between depressive and control subjects using zero level for decision making: a subject is identified as depressive by positive and nondepressive by negative SASI value. 

The adjustment of the SASI calculation algorithm to individual subject was performed taking into account individual alpha frequencies of the patients (Figure 2). Central frequency of the spectrum was calculated employing parabolic approximation of the spectrum in the alpha band. The reason of such approach was that the real EEG spectrum is not smooth and various side peaks can affect power distribution. At first, the frequency with the maximum spectral power *f*_max⁡_ in the region of alpha band (8–13 Hz) of the recorded EEG signal was estimated. Thereafter, the parabolic approximation was applied to the spectrum of the EEG frequency band (*f*_max⁡_ ± 2 Hz). The parabolic approximation was made by finding the coefficients of a polynomial function that fits the data in a least squares sense. The calculations were performed applying the Matlab POLYFIT tool. The maximum point of the fitted parabola *f*_*c*_ was taken as the central frequency of the spectrum for an individual subject. 

### 2.3. Higuchi's Fractal Dimension Method—HFD

HFD algorithm calculates fractal dimension of time series directly in the time domain [25]. It is based on a measure of length *L*(*k*) of the curve that represents the considered time series while using a segment of *k* samples as a unit if *L*(*k*) scales like

(7) $$\begin{array}{c} L\left(k\right)\sim k^{-\text{FD}}. \end{array}$$

The value of fractal dimension FD was calculated according to the following algorithm [25]. From given time series *X*(1), *X*(2), *X*(3),…, *X*(*N*), a new series *X*_*k*_^*m*^ is constructed as:

(8) Xkm:X(m),X(m+k),X(m+2k),…,X(m+int⁡(N−mk)·k), m=1,2,…,k.

The length *L*_*m*_(*k*) of every curve is calculated according to the formula:

(9) Lm(k)=1k[(∑i=1int⁡((N−m)/k)|X(m+ik)−X(m+(i−1)k)|)  ×N−1int⁡((N−m)/k)·k].

The length *L*(*k*) of the curve for time interval *k* is defined as average over *k* values of *L*_*m*_(*k*),*m* = 1,2,…, *k*. If *L*(*k*) scales like *L*(*k*) ~ *k* ^−FD^, the curve has fractal dimension FD, which is calculated using linear regression of graph:

(10) $$\begin{array}{c} \ln\left(L\left(k\right)\right)\sim \ln\left(\frac{1}{k}\right) \end{array}$$

according to the following formula:

(11) $$\begin{array}{c} \text{FD}=\frac{n\sum \left(x_{k}y_{k}\right)-\sum x_{k}\sum y_{k}}{n\sum \left(x_{k}^{2}\right)-{\left(\sum x_{k}\right)}^{2}}, \end{array}$$

where *x*_*k*_ = ln⁡⁡(1/*k*), *y*_*k*_ = ln⁡⁡*L*(*k*), *k* = *k*_1_,…, *k*_max⁡⁡_, and *n* denotes the number of *k*-values for which the linear regression is calculated (2 ≤ *n* ≤ *k*_max⁡_). 

FD was calculated in 2000 samples (5 s) window, and the window was shifted by 200 samples (0.5 s) with parameter *k*_max⁡_ = 50. The length of each EEG signal was 5 minutes, giving 591 FD values for a signal. FD for a subject and channel was achieved using averaging over all FD values for a signal.

To the best of our knowledge, there is no presumed decision making criteria for HFD value differentiating depressive and healthy subjects. Therefore, we selected the decision making level for differentiating depressive and nondepressive subjects as an average HFD value plus standard deviation for control group: the subjects with HFD values higher than the selected limit were identified as depressive and vice versa. 

### 2.4. Statistics

Probability of differentiation between depressive and control subjects on the bases of calculated SASI or HFD values was evaluated using Student's *t*-test for two-tailed distribution with two-sample unequal variance. The confidence level of *P* < 0.05 was considered statistically significant. 

## 3. Results 

### 3.1. SASI Method

**Figure 3:**
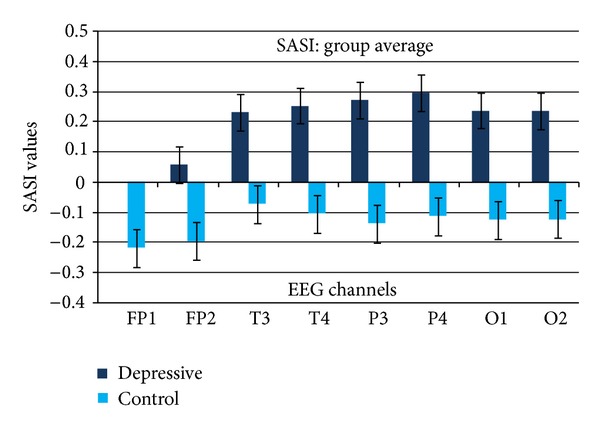
SASI values averaged over a group of depressive (DEPR) and control (CONT) subjects (*n* = 17) in various EEG channels. Vertical bars denote standard error.

Figure 3 presents calculated SASI values averaged over the groups of depressive and control subjects. The graphs indicate clearly positive average SASI values for depressive and negative for control group in all EEG channels. The most evident difference between depressive and control group appears in parietal channels. 

Numerical values of calculated parameters and statistical analysis of the results are presented in Table 1. The SASI value averaged over all EEG channels is 0.200 in the depressive and −0.136 in the control group. Standard deviation values are comparable with the values of average and even higher. Despite that, SASI method clearly differentiates depressive and healthy subjects, and statistically significant differences between the groups were revealed in all EEG channels.

**Figure 4:**
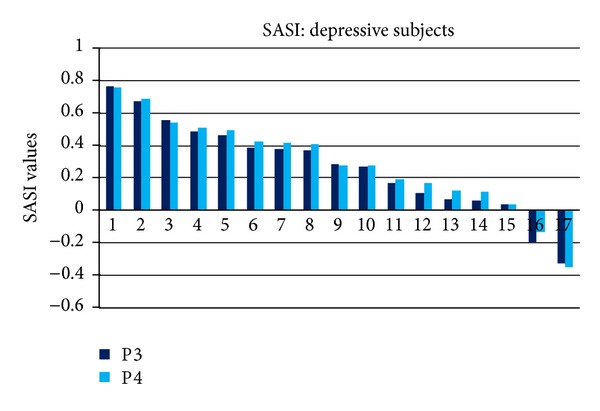
SASI values in P3 and P4 channels for individual depressive subjects.

**Figure 5:**
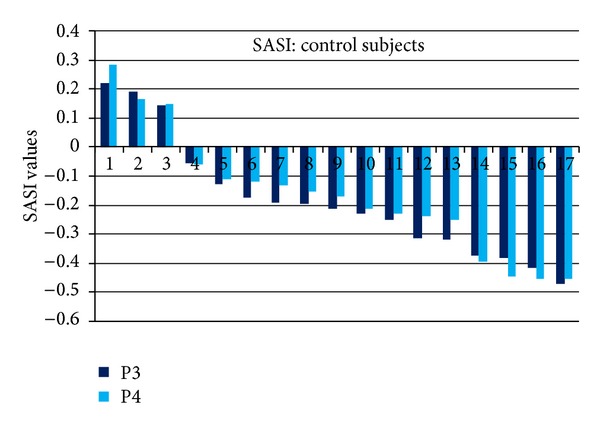
SASI values in P3 and P4 channels for individual healthy controls.

The SASI values in P3 and P4 EEG channels for depressive individual subjects are presented in Figure 4 and control subjects in Figure 5. Individual SASI values varied from −0.32 to 0.76 in depressive and from −0.47 to 0.28 in control group. Remarkable variations between individual subjects explain high values of standard deviation in Table 1. SASI values are positive for 15 depressive subjects (88%) and negative only for 2 subjects. For control group, SASI was negative for 14 (82%) and positive for 3 subjects. The rate of the subjects correctly indicated in both groups is 85%. 

SASI values, as presented in Figures 3, 4, and 5 and in Table 1, are comparable in right and left hemispheres; statistically significant difference between symmetric channels was not revealed (*P* > 0.05). 

### 3.2. HFD Method

**Figure 6:**
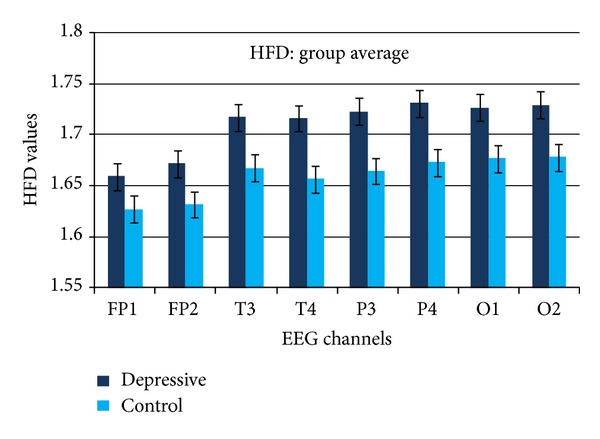
HFD values averaged over a group of depressive (DEPR) and control (CONT) subjects (*n* = 17) in various EEG channels. Vertical bars denote standard error.

Calculated HFD values averaged over all subjects are presented in Figure 6. The increase of HFD with depression is evident in all EEG channels. Numerical values of calculated HFD parameters and the results of statistical analysis are presented in Table 2. The value of depression HFD averaged over all EEG channels (1.709) is higher than in control group (1.659). The average increase of HFD values with depression is 0.05 (3%). The average standard deviations in depressive (0.0487) and control groups (0.0462) are comparable with HFD average difference between the groups (0.05). However, clear trend of increase with depression results in statistically significant differences between depressive and control groups in temporal, parietal, and occipital channels (Table 2).

**Figure 7:**
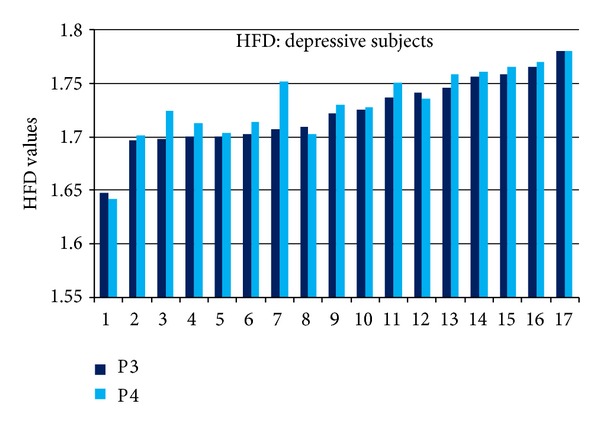
HFD values in P3 and P4 channels for individual depressive subjects.

**Figure 8:**
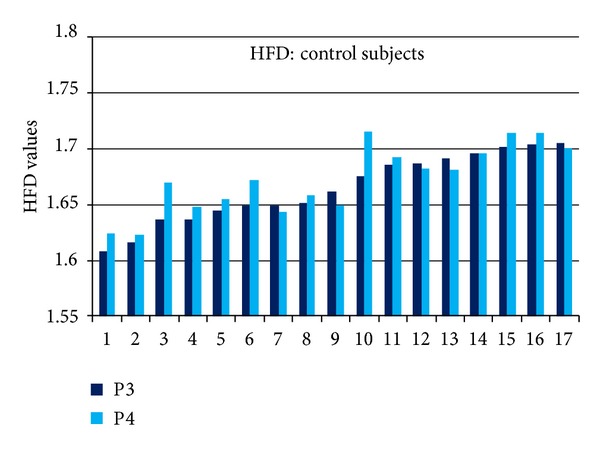
Calculated HFD values in P3 and P4 channels for individual control subjects.

The HFD values in P3 and P4 EEG channels for individual subjects in depressive group are presented in Figure 7 and in control group in Figure 8. HFD values varied from 1.64 to 1.78 in depressive and from 1.61 to 1.715 in control group. Variability between individual subjects reaches 22%. High variability between individual subjects prevails alterations of HFD related to depression (average 3%). 

The decision making level for differentiating individual subjects, determined as a mean HFD value plus standard deviation is 1.6960 in channel P3 and 1.6989 in channel P4. According to the differentiation level, 16 (94%) subjects are revealed as depressive and 1 subject as nondepressive in depressive group employing signals in channel P3. The signals in channel P4 show exactly the same result. In control group, 13 (76%) subjects are indicated as nondepressive using signals in channel P3. The signals from channel P4 indicated also 13 (76%) subjects as nondepressive; however, two subjects (10 and 13 in Figure 8) have been indicated differently using signals in channel P3 or P4. The rate of subjects correctly indicated in both groups is 85% based on analysis of signals in a single channel P3 or P4. 

Symmetry for HFD values in symmetric EEG channels of right and left hemisphere is evident at the group level (Figure 6). Some interhemispheric asymmetry of HFD values is noticeable for individuals in depressive and control groups (Figures 7 and 8). However, the difference of interhemispheric asymmetry between depressive and control group appeared statistically not significant (*P* > 0.05).

## 4. Discussion

The results presented in Figures 3 and 6 and Tables 1 and 2 demonstrate that both EEG analysis methods, SASI and HFD, clearly differentiate specific depression features in EEG. Both methods demonstrated the best results in parietal EEG channels. SASI and HFD both increased in depression. 

SASI demonstrated a remarkable increase causing change of the polarity of the parameter, while calculated HFD indicated only small increase (3%) with depression. Despite relatively small increase of HFD, the alterations were statistically significant in all EEG channels (*P* < 0.05), except frontal area. Similar relatively small but significant alteration of HFD values has been reported also in the case of other mental diseases as schizophrenia [11]. Even smaller increase in the HFD (1.3%) indicated statistically significant changes in the EEG caused by microwave exposure [26]. Different behaviour of the SASI, and HFD methods demonstrated in detection of depression can be explained by different nature of the linear and nonlinear EEG analysis. 

SASI indicated 15 (88%) subjects as depressive and HFD indicated 16 (94%) subjects as depressive in depressive group. The subject not indicated as depressive by HFD was not indicated as depressive also by SASI. SASI indicated 14 (82%) subjects as nondepressive, and HFD indicated 13 (76%) subjects as nondepressive in control group. SASI and HFD both identified the same 12 subjects as nondepressive in control group. Two subjects were identified differently by SASI and HFD. Total number of subjects 29 (85%) identified correctly in depressive and control groups was the same for SASI and HFD. The same numbers for HFD and EEG power features have been also reported for accuracy of classification (76.6%) by other authors [20]. HFD as nonlinear method demonstrated somewhat higher sensitivity for the detection of depression (94%) compared to SASI (88%).

Very high variability of SASI (Figures 4 and 5) and HFD (Figures 7 and 8) values for individual subjects (HFD varies about 22%) exceeds the difference of the measures between depressive and control groups (3% for HFD). High inter-individual variability is evident in the majority of physiological processes and measures. This factor can be considered as the disadvantage neither for SASI nor for HFD EEG analysis methods. 

The results of SASI method varies from the results of other linear methods used for detection of depression [14–16]. First of all, SASI detects the most remarkable alterations in parietal not in frontal brain region as in the case of frontal interhemispheric asymmetry [14–16]. It is also remarkable that the calculated SASI is highly similar for all symmetric channels of right and left hemispheres, whereas interhemispheric asymmetry has been reported to be a most specific feature of depressive EEG [14–16]. To explain this symmetry, we have to keep in mind that SASI bases on relative difference of powers of two EEG frequency bands in the same EEG channel. The relative difference does not depend on the absolute power levels. Therefore, a possible asymmetry of EEG powers in different hemispheres does not affect the relative distribution of powers between two frequency bands in one channel. Consequently, the interhemispheric symmetry of SASI does not contradict possible interhemispheric asymmetry of EEG power. 

The result achieved with HFD method is not directly comparable to studies in depression EEG reported by other authors [20, 21]. The authors reported only the results of classification of EEG in depression by genetic algorithm [20]. HFD calculations were used only as an input to classification technique, and information about HFD values themselves was not provided [20]. In another study, Ahmadlou et al. calculated HFD only in frontal area and revealed significant difference between depressive and control groups only in EEG beta and gamma bands [21]. The HFD is known to be sensitive to noise and frequency band [9]. Therefore, the results of studies performed on signals of different frequency bands are not comparable. Our results can be most likely compared to full-band EEG results in the study by Ahmadlou et al., where discrimination between depressive and control group was also not successful in the frontal area. Unfortunately, Ahmadlou et al. did not consider HFD in the parietooccipital area, where the best discrimination occurred in this study. 

The SASI method revealed significant difference between depression and normal EEG in all EEG channels (Table 1), whereas the HFD method detected significant difference in temporal, parietal, and occipital channels (Table 2). The SASI appears also less sensitive in frontal area (Figure 3, Table 1). The reason of lower sensitivity of the methods in frontal area is most probably related to higher variability of the frontal EEG. The elevated variability of the frontal signals behaves as an additional noise. Higuchi's algorithm is known to be sensitive to the noise level [6]. Therefore, HFD method, as more sensitive to noise loses its sensitivity in frontal EEG channels. The sensitivity of SASI method, as a more simple and robust approach, appears to be not so strongly affected by the noise. On the other hand, in the parietal area where the signals are more stable, the HFD method demonstrated better discrimination *P* = 9.05 × 10^−6^ (Table 2) compared to SASI *P* = 8.04 × 10^−5^ (Table 1).

Both methods, the SASI and HFD, have some common features in detecting depression. First, the methods discriminate depression most effectively in parietal brain area. Second, the methods do not indicate statistically significant difference in hemispheres' asymmetry between depressive and control groups, as neither of the methods depends on absolute level of the EEG signal. For that reason, the undiscovered interhemispheric asymmetry using SASI and HFD methods does not contradict possible interhemispheric asymmetry of EEG power discovered using other EEG analysis methods. 

## 5. Conclusions

Both methods, SASI and HFD, provide clear distinction of depressive features in a single-channel EEG and reveal statistically significant differences between depressive and control groups. Advantages of both SASI and HFD methods, compared to other EEG analysis methods, are

- detection of depression employing single-channel EEG signal;
- simple and fast algorithms for calculations.

The advantage of SASI method, compared to HFD, is zero level of decision making providing simple positive-negative differentiation between depressive and control subjects. Expected advantage of HFD as a nonlinear method was higher sensitivity, compared to SASI, demonstrated in the posterior brain area with lower natural variability of the EEG signal. 

The disadvantage of HFD, compared to SASI, is more complicated discrimination between individual depressive and control subjects due to relatively small difference between the HFD values. 

The main disadvantage of both methods, SASI and HFD, is that various brain disorders other than depression can cause similar alterations of the measures. Therefore, further investigations of both methods on independent and larger databases are highly required. 

## Figures and Tables

**Table 1:** Calculated parameters for SASI in various EEG channels: group averaged values, standard deviations, and *P* values differentiating depressive and control group.

EEG channel	FP1	FP2	T3	T4	P3	P4	O1	O2
Depressive group								
Average	0.0180	0.0573	0.2331	0.2536	0.2712	0.2951	0.2373	0.2371
St. dev.	0.2790	0.2546	0.2902	0.2143	0.2802	0.2564	0.2887	0.2132
Control group								
Average	−0.2181	−0.1949	−0.0726	−0.1038	-0.1370	−0.1136	−0.1257	−0.1236
St. dev.	0.1905	0.2754	0.2282	0.2397	0.2141	0.1977	0.2401	0.2573

*P* values	0.0074	0.0082	0.0071	0.0032	8.04 × 10^−5^	6.99 × 10^−5^	0.0038	0.0034

**Table 2:** Calculated parameters for HFD in various EEG channels: group averaged values, standard deviations, and *P* values differentiating depressive and control group.

EEG channel	FP1	FP2	T3	T4	P3	P4	O1	O2
Depressive group								
Average	1.6690	1.6715	1.7171	1.7164	1.7229	1.7310	1.7268	1.7291
St. dev.	0.0670	0.0752	0.0525	0.0645	0.0329	0.0341	0.0322	0.0309
Control group								
Average	1.6272	1.6316	1.6677	1.6565	1.6647	1.6728	1.6767	1.6781
St. dev.	0.0505	0.0402	0.0723	0.0600	0.0313	0.0261	0.0421	0.0426

*P* values	0.1295	0.0658	0.0354	0.0086	9.05 × 10^−6^	9.56 × 10^−6^	0.0005	0.0004
